# Editorial: Advances in bioelectronics and stimulation strategies for next generation neuroprosthetics

**DOI:** 10.3389/fnins.2022.1116900

**Published:** 2023-01-10

**Authors:** Tianruo Guo, Yao-chuan Chang, Luming Li, Socrates Dokos, Liming Li

**Affiliations:** ^1^Graduate School of Biomedical Engineering, The University of New South Wales Sydney, Sydney, NSW, Australia; ^2^Institute of Bioelectronic Medicine, Feinstein Institutes for Medical Research, Manhasset, NY, United States; ^3^Medtronic PLC, Minneapolis, MN, United States; ^4^National Engineering Research Center of Neuromodulation, School of Aerospace Engineering, Tsinghua University, Beijing, China; ^5^Precision Medicine and Healthcare Research Center, Tsinghua-Berkeley Shenzhen Institute, Tsinghua University, Shenzhen, China; ^6^IDG/McGovern Institute for Brain Research, Tsinghua University, Beijing, China; ^7^School of Biomedical Engineering, Shanghai Jiao Tong University, Shanghai, China

**Keywords:** non-invasive stimulation, optimal implantation location, stimulus parameter identification, neural ultrastructure, neuromodulation

Recent technological advances have expanded our understanding of how artificial stimulation interacts with the living nervous system (Riva and Micera, 2021; Saha et al., 2021; Walter, 2021; Ahmed et al., 2022; Chen et al., 2022; Yao et al., 2022). This Research Topic, contributed to by electrophysiologists, biomedical engineers, computational neuroscientists, and neuropsychologists, provides a state-of-the-art overview of advances in neural stimulation technologies, ranging from recent progress in functional electrical stimulation (FES) to new understanding of how weak electric field (EF) stimulation affects cellular properties. The submissions to this Research Topic can be grouped into four key areas: (A) non-invasive stimulation, (B) optimal implantation location, (C) advances in stimulus parameter identification, and (D) the influence of neural ultrastructures under EF. It is helpful to provide four quotes from Sun Tzu's *The Art of War*^1^ to represent the theme of each section:

## “The greatest victory requires no bleeding” — Chapter. Strategic attack 《孙子兵法·谋攻篇》

Significant research efforts have focused largely on developing non-invasive or minimally invasive stimulation techniques (Boes et al., 2018; Sun et al., 2018; Guo et al., 2020; Su et al., 2021, 2022; Lu et al., 2022; Ren et al., 2022). For example, transcranial direct current stimulation (tDCS) allows reversible region-specific modulation (Filmer et al., 2014). Liu S. et al. offer valuable insights into how prefrontal tDCS affects the attention bias by detecting electroencephalographic characteristics in response to rest and emotional oddball tasks. In this clinical study, tDCS caused increased brain neural activities related to emotion regulation and distinguished electrical signatures following positive targets and negative distracters, indicating great potential for tDCS in the treatment of depression. Another non-invasive neuromodulatory technique is that of trans-spinal direct current stimulation (tsDCS). Song and Martin found that cathodal tsDCS can selectively target voltage-dependent calcium channels to modulate motoneuron activity, informing therapeutic treatment strategies to achieve rehabilitation goals after injury; in particular, to increase muscle force.

In addition, motor imaginary (MI)-based brain-computer interface (BCI) must overcome multiple issues to be commercially usable, especially related to signal quality and subject-variation (Singh et al., 2021). Sensory threshold somatosensory electrical stimulation (st-SES) has been recently used to guide participants in motor imaginary tasks (Corbet et al., 2018; Vidaurre et al., 2019; Zhang et al., 2022). Chen et al. suggest that st-SES can only improve brain-switch BCI performance in those subjects with higher classification accuracy (high performers) in discriminating the MI condition from rest. Moreover, they showed that st-SES influences functional connectivity of the fronto-parietal network, but through different frequency bands for different subjects. These findings can potentially help to optimize guidance strategies to adapt to different types of MI-BCI users.

## “Choose the favorable terrain before the war starts” — Chapter. Terrain 《孙子兵法·地形篇》

An optimal implantation region improves not only stimulation performance but also the long-term stability of implantable microelectrodes, as well as reducing side effects (Wang et al., 2020; Song et al., 2022; Zhao et al., 2022). Urdaneta et al. describe a somatosensory cortex layer-dependent long-term stability in intracortical microstimulation. Their results suggest a more consistent stimulation efficiency and less foreign body response when the electrodes were implanted in L4 and L5 of the somatosensory cortex, indicating the critical role of interface depth in the design of chronic implants. Another example of optimizing electrode placement is for electroconvulsive therapy (ECT) for severe treatment-resistant depression. Steele et al. proposed a fronto-medial ECT electrode placement that would maximize the EF in specific sagittal brain regions, whilst minimizing EF in sub-regions of the bilateral hippocampi. Such outcomes suggest electrode location can significantly reduce cognitive and non-cognitive side-effects.

## “Fight smarter not harder” — Chapter. Military dispositions 《孙子兵法·军形篇》

Programming nerve stimulation setting is challenging and time consuming due to the huge number of possible stimulus parameter combinations (O'Doherty et al., 2011; Li et al., 2013; Yan et al., 2016; Jia et al., 2018; Guo et al., 2019; Muralidharan et al., 2020; Song et al., 2020; Zhang et al., 2020; Chang et al., 2022). In terms of FES, several “smart” strategies have been used to improve its effectiveness and acceptability. Xu et al. have introduced a control strategy for FES parameter selection, based on a direct transfer function using surface electromyography (sEMG) features and joint angles as inputs. A similar idea has been used historically in other neuromodulation fields since various stimulation parameters have been shown to evoke distinct neurological and physiological responses. Conversely, elicited physiological effects, both for targeted and untargeted neurons, can guide stimulus parameter tuning for many neural systems, including the brain (Qian et al., 2016; Chen et al., 2020, 2022), spinal cord (Verrills et al., 2016), vagus nerve (Chang et al., 2020), and the retina (Guo et al., 2018). In another example, Dong et al. have proposed a walking assistance system with adaptive algorithm to support FES therapy. As the stimulation sequence is tailored to the individual need based on real-time gait phase, healthy subjects are able to achieve better treadmill performance for various speed conditions. A similar adaptive idea has also been successfully adopted in clinical neuromodulation therapies to improve effectiveness, such as deep brain stimulation (Bocci et al., 2021) and spinal cord stimulation (Schultz et al., 2012), utilizing chosen physiological indices.

## “Know the enemy, know yourself” — Chapter. Strategic attack 《孙子兵法·谋攻篇》

Electrical stimulation performance cannot be significantly improved by only optimizing the device in isolation without considering the biophysical complexity of the target nerve system (Abbasi and Rizzo, 2021; Ahmed et al., 2022; Italiano et al., 2022). Sophisticated computational models have been widely used in predicting the role of tissue or neural or ultra-structures under EF (Guo et al., 2014, 2016; Yang et al., 2018; Bai et al., 2019a; Dokos and Guo, 2020). Liu(a) et al. have proposed a new biophysical model of myelin ultra-structures by simulating cytoplasmic channels in the myelin sheath as a low-impedance route, while previous models approximate the myelin sheath as an insulation layer (Schwarz and Reid, 1995; Bean, 2007; Ge et al., 2020). Using this model, Liu(b) et al. further investigated how cytoplasmic channels affect EF across the myelin sheaths, concluding that the externally applied EF can control myelin growth. These new findings indicate the possibility of using electrical modulation to treat degenerative neural diseases. Neurodegenerative progression can affect the neuroprosthetic performance (Rattay et al., 2001; Hilker et al., 2005; Loizos et al., 2018; Ly et al., 2022). Croner et al. have investigated the differential performance of cochlear stimulation in a cochlea with intact and degenerating spiral ganglion neurons (SGNs) using a biophysically detailed computational model of the human cochlea (Bai et al., 2019b). Their study identified the increased activation of neurons in unintended areas, and an insensitive neural response to various apical electrode settings when degenerating SGNs were stimulated. This study also suggested that stimulation thresholds are unlikely to be a good indicator of neural health, since degenerating SGNs showed both an increase and decrease in current threshold depending on the initial stimulation site.

## Author's note

The Chinese characters shown in the Editorial refer to the origin of each subtitle in the ancient literature The Art of War by Sun Tzu.

## Author contributions

All authors listed have made a substantial, direct, and intellectual contribution to the work and approved it for publication.

## Footnotes

^1^*The Art of War* is an ancient Chinese military treatise written in 5th century BC. It remains the most influential strategy text used in military thinking, business tactics, legal strategy, politics, sports, lifestyles and beyond.
